# gtrellis: an R/Bioconductor package for making genome-level Trellis graphics

**DOI:** 10.1186/s12859-016-1051-4

**Published:** 2016-04-18

**Authors:** Zuguang Gu, Roland Eils, Matthias Schlesner

**Affiliations:** Division of Theoretical Bioinformatics (B080), German Cancer Research Center (DKFZ), Im Neuenheimer Feld 280, 69120 Heidelberg, Germany; Heidelberg Center for Personalized Oncology (DKFZ-HIPO), German Cancer Research Center (DKFZ), Im Neuenheimer Feld 280, 69120 Heidelberg, Germany; Department for Bioinformatics and Functional Genomics, Institute for Pharmacy and Molecular Biotechnology (IPMB) and BioQuant Center, Heidelberg University, Im Neuenheimer Feld 267, 69120 Heidelberg, Germany

**Keywords:** Software, Trellis graphics, Genomic data visualization

## Abstract

**Background:**

Trellis graphics are a visualization method that splits data by one or more categorical variables and displays subsets of the data in a grid of panels. Trellis graphics are broadly used in genomic data analysis to compare statistics over different categories in parallel and reveal multivariate relationships. However, current software packages to produce Trellis graphics have not been designed with genomic data in mind and lack some functionality that is required for effective visualization of genomic data.

**Results:**

Here we introduce the *gtrellis* package which provides an efficient and extensible way to visualize genomic data in a Trellis layout. *gtrellis* provides highly flexible Trellis layouts which allow efficient arrangement of genomic categories on the plot. It supports multiple-track visualization, which makes it straightforward to visualize several properties of genomic data in parallel to explain complex relationships. In addition, *gtrellis* provides an extensible framework that allows adding user-defined graphics.

**Conclusions:**

The *gtrellis* package provides an easy and effective way to visualize genomic data and reveal high dimensional relationships on a genome-wide scale. *gtrellis* can be flexibly extended and thus can also serve as a base package for highly specific purposes. *gtrellis* makes it easy to produce novel visualizations, which can lead to the discovery of previously unrecognized patterns in genomic data.

**Electronic supplementary material:**

The online version of this article (doi:10.1186/s12859-016-1051-4) contains supplementary material, which is available to authorized users.

## Background

Trellis graphics are a visualization method that splits data by one or more conditional variables, displays subsets of the data in parallel panels and arranges the panels in a grid-style (the trellis) [1]. Each panel contains the same type of graphics, and thus the Trellis graphics enable direct comparison of the same statistics over different categories. Trellis graphics have broad applications in genomic data analysis. For example, chromosomes can be used as categorical variable to divide the genome in a natural manner. Visualizing read coverage from whole genome sequencing data by means of Trellis graphics gives a clear overview of large-scale genomic alteration [2].

In the R programming environment, for example the *lattice* [3] and *ggplot2* [4] packages provide rich functionality for making Trellis graphics. However, efficient visualization of genomic data in Trellis graphics requires the following functionalities which are, to our knowledge, in this combination not provided by any currently available package. First, in standard Trellis graphics, all panels have the same width. When mapping panels to chromosomes, short chromosomes are extended with empty areas, which makes usage of the plotting space inefficient. Thus layouts with more flexible panel width are required for genomic data visualization. Second, genomic data is multi-dimensional and it is often necessary to visualize several data tracks simultaneously to facilitate the identification of correlations between different types of information. Third, genome data analysis can greatly benefit from highly specialized visualizations which require that the user can easily add user-defined graphic elements to the panels.

Here, we present the *gtrellis* package which provides a flexible and efficient way to arrange genomic categories as Trellis layout. The flexible layouts supported by *gtrellis* ensure efficient use of plotting space so that the areas for genomic categories are larger and more details can be observed compared to standard Trellis layout. The package supports multiple-track visualization, which makes it easy to explain genomic data from different aspects. *gtrellis* provides an extensible framework that allows the addition of user-defined graphics to the panels. Based on this feature, the possible visualizations are not restricted by the package itself and the package can serve as a base that can be extended for genomic analyses with highly specific purposes. In this paper, we give a detailed explanation of the functionality provided by *gtrellis* and demonstrate the power of the package with two real-world examples. We believe that *gtrellis* can greatly help to visualize genomic data more efficiently and thus to discover previously unrecognized patterns in genomic data.

## Implementation

*gtrellis* abstracts the generation of Trellis graphics into two independent steps: i) creation of the global layout; and ii) adding graphics to the panels afterwards.

### Creation of global layout

**Fig. 1:**
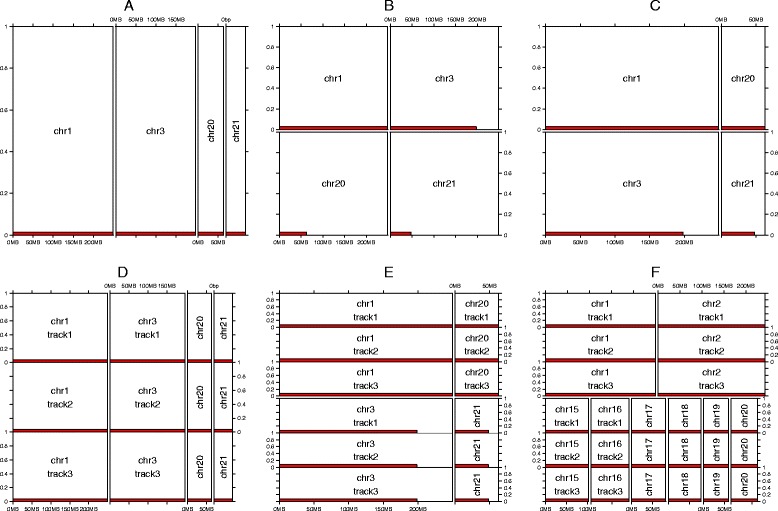
Different layout styles by *gtrellis.* **a** Default style with all chromosomes sorted in karyotypic order and arranged in one row; (**b**) Standard Trellis layout in which all panels have the same width; (**c**) Optimized layout which arranges chromosomes with similar length into the same column; (**d, e**) Layouts for parallel visualization of three data tracks with chromosomes arranged in one row (**d**) or two rows (**e**); (**f**) Non-rectangular layout without vertical alignment of panels in different rows for compact arrangement of chromosomes. In each panel, red rectangles represent the length of the corresponding chromosomes

*gtrellis* provides a simple and flexible way to initialize the global Trellis layout. In the initialization step, panels corresponding to genomic categories are allocated to proper positions in the plotting area. Positions and style of the Trellis layout can be adjusted either as traditional style or as an improved style for efficient genome data visualization. Figure 1 illustrates several styles for the global layout that can be produced by *gtrellis*. To make a clear comparison between different layout styles, two long chromosomes (human chromosome 1 and 3) and two short chromosomes (human chromosome 20 and 21) are selected. Figure 1a is the default style in which all chromosomes are arranged in one row in karyotypic order, and the panel width is proportional to each chromosome’s length. Such one-row style layout is broadly used by a lot of packages that focus on genomic data analysis (e.g. showing genome-wide read coverage or copy number variation from whole genome sequencing data). However, the drawback is that with increasing number of chromosome, the plotting region for each chromosome will shrink, limiting the possible resolution of visualized features. Figure 1b illustrates a standard Trellis layout. It is obvious that there are huge empty areas in the panels of chromosome 20 and 21, which makes the layout inefficient. In Fig. 1c, the traditional Trellis layout is improved so that chromosomes with similar length are put in the same column, resulting in a much better usage of plotting space compared to Fig. 1a and b.

To support the identification of correlations in genomic data, *gtrellis* allows creating multiple tracks for each genomic category. In each track, one specific type of information is displayed. Figure 1d and e show layouts with three tracks for each chromosome, arranging the chromosomes in either one or two rows.

Finally, *gtrellis* supports a non-rectangular layout that compactly arranges chromosomes without alignment of panels between different rows (Fig. 1f). This layout makes highly efficient use of the plotting space, but loses the direct comparability of positions for different categories across rows.

In most cases, chromosomes are used as the category variable when making genome-level plots. However, *gtrellis* supports more general genomic categories by specifying a list of background regions. Additional file 1 illustrates transcript models for three genes (TP53, TP63 and TP73) by taking gene as the genomic category, and arranges them in two types of Trellis layout.

### Adding graphics

After initialization of the layout, each panel in one specific track and one specific genomic category serves as an independent plotting region. Graphics can be added into the panel afterwards. *gtrellis* opens an application programming interface (API) that allows users to customize their own graphics and put it into each panel. This type of design enhances the flexibility of the package greatly, because the types of graphics that can be plotted are not restricted by the package itself. In *gtrellis*, the *add_track()* function adds graphics to one track in batch mode, and sequential calling of *add_track()* adds graphics to multiple tracks. *add_track()* receives a *panel_fun* argument which is a user-defined function to be applied to every panel. The input genomic data for the panel function is either a data frame or a *GRanges* object which covers most of the formats used in genomic data analysis. When *add_track()* is executed, the panel function iterates over all genomic categories and automatically extracts the subset of data which belongs to the current genomic category for the panel. The following code gives an example for adding points in one track.



*add_track()* can also be applied to an individual panel by specifying the index of the corresponding category and track. This allows customization of specific panels in the plot.



In order to simplify customization of the track, *gtrellis* provides the following five functions *add_points_track()*, *add_lines_track()*, *add_rect_track()*, *add_segments_track()* and *add_heatmap_track().* These functions add commonly used graphic elements to the track without the need to define an own panel function.

Since layout creation and adding graphics are two independent steps in *gtrellis*, different layouts can be used without changes in the code which is used for adding graphics (see example in Additional file 1 and package vignette). This strategy results in modular, flexible and reusable plotting code.

## Results and discussion

### Comparison to other packages

*gtrellis* has been specifically designed for effective visualization of genomic data. While several existing packages provide functionalities to generate Trellis graphics, none of them can fulfil all particular requirements of genomic data visualization. Table 1 shows a comparison between existing packages and *gtrellis* with respect to these functionalities. *ggplot2* and *lattice* are able to arrange genomic categories (e.g. chromosomes) into different panels as a standard Trellis layout. However, each panel has the same width, so that short chromosomes are extended by empty space. *ggbio* [5] supports a layout with panel width proportional to the chromosome length when making a Manhattan plot. The limitation is, however, that all chromosomes can only be put in the same row. In standard figure formats this results in small plotting regions per chromosome, meaning that several patterns may remain invisible (Additional file 2 compares the standard Manhattan plot and the enhanced plot by *gtrellis*). *lattice* can be enhanced by *latticeExtra* [6] to support unequal panel width, but as discussed later, it does not support multiple-track visualization. In contrast, *gtrellis* provides efficient layouts to arrange chromosomes on the page, including a unique layout that arranges the chromosomes in one row compactly (Fig. 1f) to maximize the utilization of the plotting space. *Gviz* [7] is generally designed to visualize smaller genomic regions. With the *grid* graphic system, it is possible to arrange multiple regions into one page through *Gviz*, but there are several limitations: 1) The y-axes are repeated in all sub-plots which is unnecessary; 2) Scales on the x-axes are not consistent in sub-plots which aggravates comparisons across sub-plots, and the panel widths for all sub-plots are identical regardless of the lengths of the corresponding regions, which results in different scales and makes it difficult to compare regions directly. Multiple track visualization greatly helps to identify correlations between different sources of information. It is well supported by *gtrellis*, while *ggplot2* and *lattice* do not support multiple-track visualization. *Gviz* and *ggbio* can integrate multiple sources of information for a single genomic region, but not for chromosome- or genome-wide visualizations. Finally, *gtrellis* provides an open API to allow user-defined graphics, while *ggplot2*, *lattice*, and *Gviz* only provide fixed types of graphics without simple methods for further customization.

**Table 1:** Comparison of functionalities for genomic data visualization between *gtrellis* and existing packages

	*ggplot2*	*lattice*	*Gviz*	*ggbio*	*gtrellis*
Multiple genomic categories	yes	yes	limited	yes	yes
Flexible panel width	no	limited	no	no	yes
Multiple-track visualization	no	no	yes	yes	yes
Plotting of user-defined graphics	limited	limited	limited	no	yes

### Example applications

In this section we provide two examples that demonstrate the extensibility and power of *gtrellis* to effectively visualize different types of genomic data and reveal relations between different sources of information. Furthermore, Additional file 2 demonstrates how enhanced Manhattan plots can reveal patterns in the distribution of significant SNPs in the genome.

**Fig. 2:**
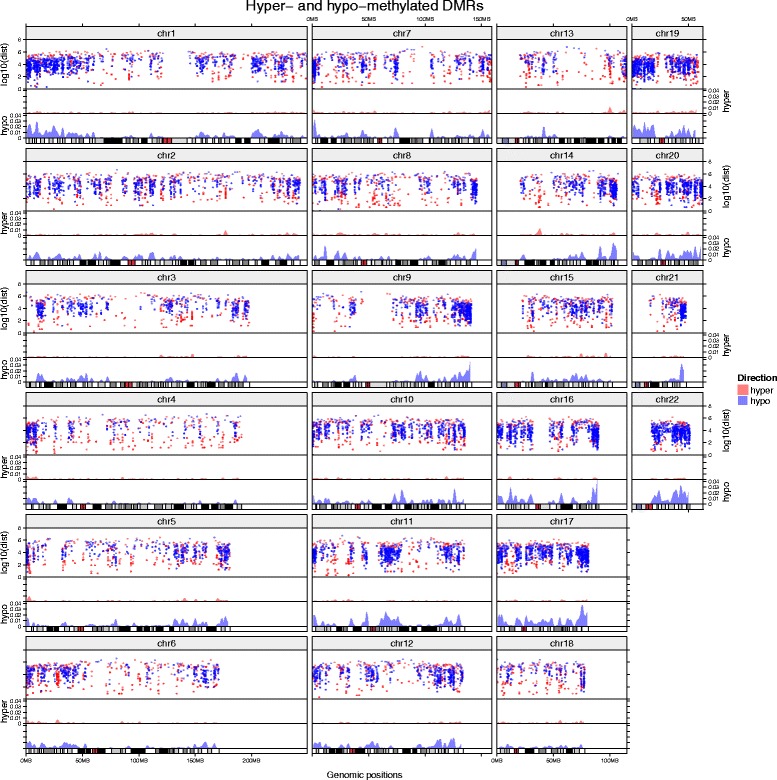
Visualizing differentially methylated regions. Differentially methylated regions (DMRs) between Burkitt lymphomas and germinal center-derived B-cells are illustrated in rainfall plots to visualize the genomic distribution and identify localized clusters. There are five tracks for each chromosome (from top to bottom): (i) chromosome names; (ii) rainfall plots for both hyper-methylated DMRs and hypo-methylated DMRs; (iii) genomic density for hyper-methylated DMRs; (iv) genomic density for hypo-methylated DMRs; and (v) ideograms

The first example (Fig. 2) illustrates the distribution of genomic regions which are differentially methylated (DMRs) in Burkitt lymphomas compared to germinal center B cells [8]. A DMR is a genomic interval in which methylation levels at CpG sites are significantly different between disease and control samples. A DMR is designated hyper-methylated if the methylation is higher in disease than in control samples and hypo-methylated if the methylation is lower in disease samples.

In Fig. 2, rainfall plots are used to visualize the distribution of DMRs in the genome. Rainfall plots are particularly useful to identify clusters of DMRs. In the rainfall plot, each dot represents a DMR. The x-axis corresponds to the genomic coordinate, and the y-axis corresponds to the minimal distance (log10 transformed) of the DMR to its two neighbouring DMRs. A cluster of DMRs will appear as a “rainfall” in the plot. However, if the amount of DMRs in a cluster is high, dots will overlap, and direct assessment of the number and density of DMRs in the cluster will be impossible. To overcome this limitation, additional tracks are added which visualize the genomic density of DMRs (defined as the fraction of a genomic window that is covered by DMRs), separated into tracks for hyper- and hypo-methylated DMRs (two tracks in Fig. 2 labelled with “hyper” and “hypo” on the y-axes). It becomes apparent that both hyper- and hypo-methylated DMRs form clusters throughout the genome, and that the genomic density of hypo-methylated clusters is considerably higher than the genomic density of hyper-methylated clusters. Finally, a track with chromosome names and a track that contains ideograms to help to localize DMR clusters on the chromosomes are added. The source code to generate Fig. 2 can be found in Additional file 3.

**Fig. 3:**
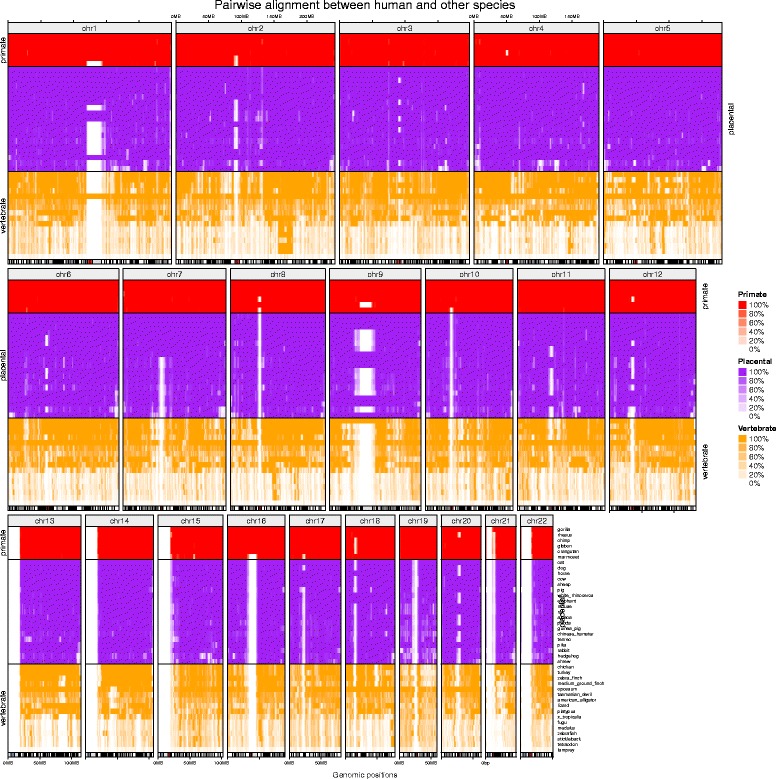
Visualizing genomic conservation between human and 41 other species. There are five tracks for each chromosome (from top to bottom): (i) chromosome names; (ii) primates; (iii) placentals; (iv) vertebrates; and (v) ideograms. The human genome has been divided into 2 MB windows, and for each window the fraction that can be aligned to the corresponding species is plotted as heatmap. The compact non-rectangular layout has been chosen to optimize plotting space utilization for the genome-wide visualization

The second example (Fig. 3) shows conservation between the human genome and 41 other species [9]. The human genome is segmented into 2 MB windows and the fraction of each window that that can be aligned to the genome of the respective species is calculated. The 41 species are categorized into primates (6 species), placentals (19 species) and vertebrates (16 species), and each category is visualized as a single track. In each track, the order of species is calculated by hierarchical clustering of the fraction values from all chromosomes. Chromosomes are arranged in a non-rectangular layout for most efficient plotting space utilization, and fraction values are displayed as heatmap. Figure 3 clearly reveals different patterns of conservation between the human genome and the other species. Source code to generate Fig. 3 can be found at Additional file 3.

## Conclusions

The *gtrellis* package provides easy and flexible methods for producing Trellis graphics that effectively visualize genomic data. The separation of layout creation and adding of graphics provides a modular way for users to design their plots. The capability to plot multiple tracks greatly enhances the visualization of multi-dimensional genomic data and improves the detection of patterns and correlations within the data.

## Availability and requirements

**Project name:***gtrellis.*

**Project home page:**http://www.bioconductor.org/packages/devel/bioc/html/gtrellis.html.

**Operation systems:** Platform independent.

**Programming language:** R (> = 3.2.0).

**License:** GPL (> = 2).

**Restrictions to use by non-academics:** None.

## Additional files

Additional file 1: Visualization of multiple transcripts for TP53, TP63 and TP73. (ZIP 79 kb) Additional file 2: Enhanced Manhattan plot. (ZIP 452 kb) Additional file 3: Data and source code for producing Figs. 2 and 3. (ZIP 1464 kb)

## Abbreviations

API: application programming interface
DMR: differentially methylated region
