# Supplementary material for: gtrellis: an R/Bioconductor package for making genome-level Trellis graphics
Source: BMC Bioinformatics. 2016 Apr 18;17:169. doi: 10.1186/s12859-016-1051-4 (PMC4835841; doi:10.1186/s12859-016-1051-4)
Supplement: Additional file 1: — Visualization of multiple transcripts for TP53, TP63 and TP73. (ZIP 79 kb) [file 12859_2016_1051_MOESM1_ESM.zip › supplS1.html]

Supplmentary S1. Visualize multiple transcripts for TP53, TP63 and TP73


# Supplmentary S1. Visualize multiple transcripts for TP53, TP63 and TP73

**Author**: Zuguang Gu ( z.gu@dkfz.de )

**Date**: 2016-03-04

---

In **gtrellis**, genomic categories are not restricted in chromosomes. It can be any kind,
such as genes, as long as the background ranges are specified.

In following example, we put three genes in one row and draw their transcripts afterwards.

In **gtrellis** package, `tp_family.RData` contains transcripts models for TP53, TP63 and TP73.
First we calculate the ranges for the three genes.

```
library(gtrellis)
load(system.file("extdata", "tp_family.RData", package = "circlize"))
df = data.frame(gene = names(tp_family),
    start = sapply(tp_family, function(x) min(unlist(x))),
    end = sapply(tp_family, function(x) max(unlist(x))))
df
```

```
##      gene     start       end
## TP73 TP73   3569084   3652765
## TP63 TP63 189349205 189615068
## TP53 TP53   7565097   7590856
```

Since multiple transcripts are plotted stack by stack, the maximum number of transcripts for the three genes
are calculated and it will be set as the maximum value on y-axis.

```
# maximum number of transcripts
n = max(sapply(tp_family, length))
n
```

```
## [1] 17
```

`df` contains ranges for the three genes and it can be passed to `gtrellis_layout()` for initializing the Trellis layout.
Since Trellis layout will be changed in later part of this document, the code for adding graphics are wrapped into a function
for repeatitive use.

```
gtrellis_layout(data = df, n_track = 1, track_ylim = c(0.5, n+0.5), 
    track_axis = FALSE, add_name_track = TRUE, xpadding = c(0.05, 0.05), 
    ypadding = c(0.05, 0.05))
plot_transcripts_model = function(tp_family) {
    add_track(panel_fun = function() {
        gn = get_cell_meta_data("name")
        tr = tp_family[[gn]] # all transcripts for this gene
        for(i in seq_along(tr)) {
            # for each transcript
            current_tr_start = min(tr[[i]]$start)
            current_tr_end = max(tr[[i]]$end)
            grid.lines(c(current_tr_start, current_tr_end), c(n - i + 1, n - i + 1), 
                default.units = "native", gp = gpar(col = "#CCCCCC"))
            grid.rect(tr[[i]][[1]], n - i + 1, tr[[i]][[2]] - tr[[i]][[1]], 0.8,
                default.units = "native", just = "left", 
                gp = gpar(fill = "orange", col = "orange"))
        }
    })
}
plot_transcripts_model(tp_family)
```

Next we change the layout into one-column layout. Now the coordinate for all three genes are changed
to the relative positions to TSS because all three genes are aligned by their TSS on the plot. In following
example, these 'magic numbers' are positions of the TSS of corresponding genes.

```
# TP53 is on reverse strand
tp_family$TP53 = lapply(tp_family$TP53, function(df) {
        data.frame(start = 7590856 - df[[2]],
                   end = 7590856 - df[[1]])
    })
tp_family$TP63 = lapply(tp_family$TP63, function(df) {
        data.frame(start = df[[1]] - 189349205,
                   end = df[[2]] - 189349205)
    })
tp_family$TP73 = lapply(tp_family$TP73, function(df) {
        data.frame(start = df[[1]] - 3569084,
                   end = df[[2]] - 3569084)
    })
df = data.frame(gene = names(tp_family),
    start = sapply(tp_family, function(x) min(unlist(x))),
    end = sapply(tp_family, function(x) max(unlist(x))))
df
```

```
##      gene start    end
## TP73 TP73     0  83681
## TP63 TP63     0 265863
## TP53 TP53     0  25759
```

Since only the layout is changed here, `plot_transcripts_model()` can be directly applied here.

```
n = max(sapply(tp_family, length))
gtrellis_layout(data = df, n_track = 1, ncol = 1, track_ylim = c(0.5, n+0.5), 
    track_axis = FALSE, add_name_track = TRUE, 
    xpadding = c(0.01, 0.01), ypadding = c(0.05, 0.05))
plot_transcripts_model(tp_family)
```

## Session info

```
sessionInfo()
```

```
## R version 3.2.2 (2015-08-14)
## Platform: x86_64-apple-darwin13.4.0 (64-bit)
## Running under: OS X 10.11.3 (El Capitan)
## 
## locale:
## [1] C/en_US.UTF-8/C/C/C/C
## 
## attached base packages:
##  [1] stats4    parallel  methods   grid      stats     graphics  grDevices
##  [8] utils     datasets  base     
## 
## other attached packages:
## [1] gtrellis_1.3.3       GenomicRanges_1.20.8 GenomeInfoDb_1.4.3  
## [4] IRanges_2.2.9        S4Vectors_0.6.6      BiocGenerics_0.14.0 
## 
## loaded via a namespace (and not attached):
##  [1] circlize_0.3.4      formatR_1.2.1       magrittr_1.5       
##  [4] evaluate_0.8        GlobalOptions_0.0.8 stringi_1.0-1      
##  [7] XVector_0.8.0       GetoptLong_0.1.1    rjson_0.2.15       
## [10] tools_3.2.2         stringr_1.0.0       colorspace_1.2-6   
## [13] shape_1.4.2         knitr_1.11
```
