# Supplementary material for: gtrellis: an R/Bioconductor package for making genome-level Trellis graphics
Source: BMC Bioinformatics. 2016 Apr 18;17:169. doi: 10.1186/s12859-016-1051-4 (PMC4835841; doi:10.1186/s12859-016-1051-4)
Supplement: Additional file 2: — Enhanced Manhattan plot. (ZIP 452 kb) [file 12859_2016_1051_MOESM2_ESM.zip › supplS2.html]

Supplementary S2: Enhanced Manhattan plot


# Supplementary S2: Enhanced Manhattan plot

**Author**: Zuguang Gu ( z.gu@dkfz.de )

**Date**: 2016-03-04

---

The GWAS data was downloaded from UCSC Table Browser. The parameters for downloading were:

```
clade: Mammal
genome: Human
assembly: Feb. 2009(GRCh37/hg19)
group: Phenotype and Literature
track: GWAS Catalog
table: gwasCatalog
```

The processed data is already included in the **gtrellis** package:

```
load(system.file("extdata", "gwasCatalog.RData", package = "gtrellis"))
head(gwas)
```

```
##    chr   start     end       name p-value
## 1 chr1 1005805 1005806  rs3934834   6e-07
## 2 chr1 1079197 1079198 rs11260603   4e-07
## 3 chr1 1247493 1247494    rs12103   8e-13
## 4 chr1 1723030 1723031  rs9660180   8e-07
## 5 chr1 1723030 1723031  rs9660180   6e-07
## 6 chr1 2069171 2069172   rs425277   2e-08
```

```
v = -log10(gwas[, "p-value"])
# remove outliers
q95 = quantile(v, 0.95)
v[v > q95] = q95
```

First we make the normal Manhattan plot. From the plot below, basically we can only see there are
SNPs that show high significance and on chromosome 6 there exists a cluster where the SNP density
are very high.

```
library(gtrellis)
gtrellis_layout(category = paste0("chr", 1:22), track_ylim = range(v), track_ylab = "-log10(p)")
add_points_track(gwas, v, gp = gpar(col = "#00000080"))
```

Next we adjust the layout, also we add another track which shows the number of SNPs in
5MB genomic windows. In the new layout, width for each chromosome is much wider than the previous plot,
thus, it shows very clearly for the distribution pattern of highly significant SNPs
in the genome (in the previous plot, due to the narrow plotting area for each chromosome,
the distribution of SNPs seems random). The additional track gives an exact view that SNP density is dominantly high
in a cluster on chromosome 6 and there are also many small hotspot mutation areas spreading the genome.

```
library(circlize)
# how many SNPs in every 5MB window
d = genomicDensity(gwas, 5e6)
d[, 4] = d[, 4] * 5e6

gtrellis_layout(nrow = 4, byrow = FALSE, n_track = 2, category = paste0("chr", 1:22),
    add_ideogram_track = TRUE, add_name_track=TRUE, track_ylim = c(range(v), range(d[, 4])),
    track_height = c(2, 1), track_ylab = c("-log10(p)", "#SNP"))
add_points_track(gwas, v, gp = gpar(col = "#00000080"))
add_lines_track(d, d[, 4], area = TRUE, gp = gpar(fill = "#999999", col = NA))
```

## Session info

```
sessionInfo()
```

```
## R version 3.2.2 (2015-08-14)
## Platform: x86_64-apple-darwin13.4.0 (64-bit)
## Running under: OS X 10.11.3 (El Capitan)
## 
## locale:
## [1] C/en_US.UTF-8/C/C/C/C
## 
## attached base packages:
##  [1] stats4    parallel  methods   grid      stats     graphics  grDevices
##  [8] utils     datasets  base     
## 
## other attached packages:
## [1] circlize_0.3.4       gtrellis_1.3.3       GenomicRanges_1.20.8
## [4] GenomeInfoDb_1.4.3   IRanges_2.2.9        S4Vectors_0.6.6     
## [7] BiocGenerics_0.14.0 
## 
## loaded via a namespace (and not attached):
##  [1] formatR_1.2.1       magrittr_1.5        evaluate_0.8       
##  [4] GlobalOptions_0.0.8 stringi_1.0-1       XVector_0.8.0      
##  [7] GetoptLong_0.1.1    rjson_0.2.15        tools_3.2.2        
## [10] stringr_1.0.0       colorspace_1.2-6    shape_1.4.2        
## [13] knitr_1.11
```
