# Supplementary material for: gtrellis: an R/Bioconductor package for making genome-level Trellis graphics
Source: BMC Bioinformatics. 2016 Apr 18;17:169. doi: 10.1186/s12859-016-1051-4 (PMC4835841; doi:10.1186/s12859-016-1051-4)
Supplement: Additional file 3: — Data and source code for producing Figs. 2 and 3. (ZIP 1464 kb) [file 12859_2016_1051_MOESM3_ESM.zip › supplS3.html]

Supplementary S3: Produce figure 2 and figure 3 in the main manuscript


# Supplementary S3: Produce figure 2 and figure 3 in the main manuscript

**Author**: Zuguang Gu ( z.gu@dkfz.de )

**Date**: 2016-03-04

---

Load all necessary packages.

```
library(gtrellis)
library(circlize)
library(ComplexHeatmap)
```

## Produce Figure 2

The Differentially methylated data is from Supplementary table 7, Kretzmer H., *et al*. 2015 (http://www.ncbi.nlm.nih.gov/pubmed/26437030). Only the tab “DMRs\_BL.GCB” is visualized.

The DMR data is also included in this supplementary (`dmr.txt`). A column which contains
mean difference of methylation between Burkitt lymphomas and germinal center B cells
is added and a column which represents whether the DMR is hyper-methylated or hypo-methylated is added as well.

DMRs with absolute methylation difference larger than 0.3 are kept for plotting.

```
df = read.table("dmr.txt", header = TRUE, sep = "\t", stringsAsFactors=FALSE)
df = df[, -5]
df$mean_diff = df[[5]] - df[[4]]
df$direction = ifelse(df$mean_diff > 0, "hyper", "hypo")
df = df[abs(df$mean_diff) > 0.3, ]
df = df[, -(4:6)]
head(df)
```

```
##    chr   start     end direction
## 1 chr1  748461  748823      hypo
## 2 chr1  800651  800961      hypo
## 3 chr1  807945  808585      hypo
## 4 chr1  807945  808585      hypo
## 5 chr1  835124  835492      hypo
## 6 chr1 1347827 1348008     hyper
```

`df` is split into two data frames which contain hyper-methylated DMRs and hypo-methylated DMRs separately.

```
DMR_hyper = df[df$direction == "hyper", ]
DMR_hypo = df[df$direction == "hypo", ]
```

Whole genome is split by 2MB window and `genomeDensity()` from **circlize** package is used
to calculate how much each 2MB window is covered by DMRs.

```
DMR_hyper_density = genomicDensity(DMR_hyper, window.size = 2000000)
DMR_hypo_density = genomicDensity(DMR_hypo, window.size = 2000000)
head(DMR_hyper_density)
```

```
##    chr   start     end       pct
## 1 chr1       1 2000000 0.0005735
## 2 chr1 1000001 3000000 0.0015800
## 3 chr1 2000001 4000000 0.0019245
## 4 chr1 3000001 5000000 0.0007940
## 5 chr1 4000001 6000000 0.0000000
## 6 chr1 5000001 7000000 0.0009050
```

Maximum density values for both hyper- and hypo-methylated DMRs are calculated as the maximum value on y-axis
in both genomic density tracks.

```
max_density = max(c(DMR_hyper_density[[4]], DMR_hypo_density[[4]]))
```

`rainfallTransform()` from **circlize** package is used to calculate the distance to neighbouring DMRs.

```
DMR_hyper_rainfall = rainfallTransform(DMR_hyper)
DMR_hypo_rainfall = rainfallTransform(DMR_hypo)
head(DMR_hyper_rainfall)
```

```
##    chr   start     end  dist
## 1 chr1  845588  845679 49373
## 2 chr1  895052  895091     0
## 3 chr1  895052  895091     0
## 4 chr1  895300  895415     0
## 5 chr1  895300  895415     0
## 6 chr1 1347827 1348008  5418
```

Legend is generated by **ComplexHeatmap** package.

```
cm = ColorMapping(levels = c("hyper", "hypo"), colors = c("#FF000080", "#0000FF80"))
lgd = color_mapping_legend(cm, title = "Direction", plot = FALSE)
```

Next we put every thing together and make the plot.

```
gtrellis_layout(category = paste0("chr", 1:22), n_track = 3, ncol = 4, byrow = FALSE,
    track_axis = TRUE, 
    track_height = c(1, 0.5, 0.5), 
    track_ylim = c(0, 8, 0, max_density, 0, max_density),
    track_ylab = c("log10(dist)", "hyper", "hypo"),
    add_name_track = TRUE, add_ideogram_track = TRUE,
    legend = lgd, title = "Hyper- and hypo-methylated DMRs")

add_points_track(DMR_hyper_rainfall, log10(DMR_hyper_rainfall[[4]]),
    pch = 16, size = unit(0.8, "mm"), gp = gpar(col = "#FF000080"))
add_points_track(DMR_hypo_rainfall, log10(DMR_hypo_rainfall[[4]]), track = current_track(),
    pch = 16, size = unit(0.8, "mm"), gp = gpar(col = "#0000FF80"))

# track for genomic density
add_lines_track(DMR_hyper_density, DMR_hyper_density[[4]], area = TRUE, 
    gp = gpar(fill = "#FF000080", col = NA))
add_lines_track(DMR_hypo_density, DMR_hypo_density[[4]], area = TRUE,
    gp = gpar(fill = "#0000FF80", col = NA))
```

## Produce figure 3

Figure 3 visualizes how well the human genome can be aligned to other species. The pairwise alignment
is downloaded from UCSC Table Browser (http://genome.ucsc.edu/cgi-bin/hgTables). Parameters are as follows:

```
clade: Mammal
genome: Human
assembly: Feb. 2009(GRCh37/hg19)
group: Comparative Genomics
track: Primate Chain/net, Placental Chain/Net, Vertebrate Chain/Net
table: all species that correspond to 'Net'
```

The human genome is segmented by 2MB window and the percentage for
each window that is covered by aligned regions is calculated.
Processed data is stored in `conservation_to_human.RData`.

```
load("conservation_to_human.RData")
species
```

```
##                     V1         V2
## 1              lamprey vertebrate
## 2            zebrafish vertebrate
## 3               medaka vertebrate
## 4          stickleback vertebrate
## 5                 fugu vertebrate
## 6            tetraodon vertebrate
## 7         x_tropicalis vertebrate
## 8               lizard vertebrate
## 9   american_alligator vertebrate
## 10         zebra_finch vertebrate
## 11 medium_ground_finch vertebrate
## 12             chicken vertebrate
## 13              turkey vertebrate
## 14             opossum vertebrate
## 15            platypus vertebrate
## 16            marmoset    primate
## 17     tasmanian_devil vertebrate
## 18              rhesus    primate
## 19              gibbon    primate
## 20           orangutan    primate
## 21             gorilla    primate
## 22               chimp    primate
## 23              tenrec  placental
## 24            elephant  placental
## 25               shrew  placental
## 26            hedgehog  placental
## 27               panda  placental
## 28                 dog  placental
## 29                 cat  placental
## 30    white_rhinoceros  placental
## 31               horse  placental
## 32                 pig  placental
## 33                 cow  placental
## 34               sheep  placental
## 35              alpaca  placental
## 36                pika  placental
## 37              rabbit  placental
## 38     chinese_hamster  placental
## 39          guinea_pig  placental
## 40                 rat  placental
## 41               mouse  placental
```

```
head(conservation[, 1:6])
```

```
##    chr   start     end   lamprey zebrafish    medaka
## 1 chr1       1 2000000 0.1724190 0.4427220 0.2908905
## 2 chr1 1000001 3000000 0.2208160 0.5918540 0.3772455
## 3 chr1 2000001 4000000 0.1576985 0.6175870 0.3932075
## 4 chr1 3000001 5000000 0.0835485 0.3529265 0.2277490
## 5 chr1 4000001 6000000 0.0123595 0.0319930 0.0164820
## 6 chr1 5000001 7000000 0.0607365 0.2760390 0.3009390
```

Legends are defined by **ComplexHeatmap** package. Here we have three legends corresponding to primates,
placentals and vertebrates.

```
col_fun = list(primate = colorRamp2(c(0, 1), c("white", "red")),
               placental = colorRamp2(c(0, 1), c("white", "purple")),
               vertebrate = colorRamp2(c(0, 1), c("white", "orange")))
cm1 = ColorMapping(col_fun = col_fun$primate)
cm2 = ColorMapping(col_fun = col_fun$placental)
cm3 = ColorMapping(col_fun = col_fun$vertebrate)
lgd = list(color_mapping_legend(cm1, title = "Primate", plot = FALSE,
                at = c(0, 0.2, 0.4, 0.6, 0.8, 1), labels = c("0%", "20%", "40%", "60%", "80%", "100%")),
           color_mapping_legend(cm2, title = "Placental", plot = FALSE,
                at = c(0, 0.2, 0.4, 0.6, 0.8, 1), labels = c("0%", "20%", "40%", "60%", "80%", "100%")),
           color_mapping_legend(cm3, title = "Vertebrate", plot = FALSE,
                at = c(0, 0.2, 0.4, 0.6, 0.8, 1), labels = c("0%", "20%", "40%", "60%", "80%", "100%")))
```

Three tracks are created for primate, placental and vertebrate species separatedly.
Heatmaps are used to visualize conservation between species. In the `for` loop, species
are ordered by hierarchical clustering of percentage values that are merged from all chromosomes.
Additionally, the dendrogram which is from clustering is re-ordered so that species that are more similar
to human are put on the top in each heatmap.

```
type_order = c("primate", "placental", "vertebrate")
gtrellis_layout(category = paste0("chr", 1:22), n_track = 3, nrow = 3, compact = TRUE,
    track_height = table(species[, 2])[type_order], track_ylab = type_order,
    add_name_track = TRUE, add_ideogram_track = TRUE,
    track_axis = FALSE, legend = lgd, title = "Pairwise alignment between human and other species")
for(type in type_order) {
    l = species[, 2] == type

    m = conservation[, species[l, 1]]
    dend = as.dendrogram(hclust(dist(t(m)), method = "single"))
    dend = stats:::reorder.dendrogram(dend, colSums(m))
    od = order.dendrogram(dend)
    m = m[, od]

    add_heatmap_track(conservation, m, fill = col_fun[[type]])

    add_track(NULL, clip = FALSE, category = "chr22", track = current_track(), panel_fun = function(gr) {
        oxlim = get_cell_meta_data("extended_xlim")
        # to hide the ylab on the right
        grid.rect(x = unit(1, "npc") + unit(1, "mm"), width = unit(1, "cm"), just = "left", 
            gp = gpar(fill = "white", col = "white"))

        for(i in seq_len(ncol(m))) {
            grid.text(colnames(m)[i], unit(oxlim[2], "native") + unit(2, "mm"), unit((i-0.5)/ncol(m), "npc"), 
                just = "left", gp = gpar(fontsize = 6))
        }
    })
}
```

## Session info

```
sessionInfo()
```

```
## R version 3.2.2 (2015-08-14)
## Platform: x86_64-apple-darwin13.4.0 (64-bit)
## Running under: OS X 10.11.3 (El Capitan)
## 
## locale:
## [1] C/en_US.UTF-8/C/C/C/C
## 
## attached base packages:
##  [1] stats4    parallel  methods   grid      stats     graphics  grDevices
##  [8] utils     datasets  base     
## 
## other attached packages:
## [1] circlize_0.3.4       ComplexHeatmap_1.9.3 gtrellis_1.3.3      
## [4] GenomicRanges_1.20.8 GenomeInfoDb_1.4.3   IRanges_2.2.9       
## [7] S4Vectors_0.6.6      BiocGenerics_0.14.0 
## 
## loaded via a namespace (and not attached):
##  [1] dendextend_1.1.2    formatR_1.2.1       magrittr_1.5       
##  [4] evaluate_0.8        GlobalOptions_0.0.8 stringi_1.0-1      
##  [7] XVector_0.8.0       whisker_0.3-2       GetoptLong_0.1.1   
## [10] RColorBrewer_1.1-2  rjson_0.2.15        tools_3.2.2        
## [13] stringr_1.0.0       colorspace_1.2-6    shape_1.4.2        
## [16] knitr_1.11
```
